# Local and Global Effects of Climate on Dengue Transmission in Puerto Rico

**DOI:** 10.1371/journal.pntd.0000382

**Published:** 2009-02-17

**Authors:** Michael A. Johansson, Francesca Dominici, Gregory E. Glass

**Affiliations:** 1 Dengue Branch, Division of Vector-Borne Infectious Diseases, Centers for Disease Control and Prevention, San Juan, Puerto Rico, United States of America; 2 W. Harry Feinstone Department of Molecular Microbiology and Immunology, Johns Hopkins Bloomberg School of Public Health, Baltimore, Maryland, United States of America; 3 Department of Biostatistics, Johns Hopkins Bloomberg School of Public Health, Baltimore, Maryland, United States of America; University of São Paulo, Brazil

## Abstract

The four dengue viruses, the agents of dengue fever and dengue hemorrhagic fever in humans, are transmitted predominantly by the mosquito *Aedes aegypti*. The abundance and the transmission potential of *Ae. aegypti* are influenced by temperature and precipitation. While there is strong biological evidence for these effects, empirical studies of the relationship between climate and dengue incidence in human populations are potentially confounded by seasonal covariation and spatial heterogeneity. Using 20 years of data and a statistical approach to control for seasonality, we show a positive and statistically significant association between monthly changes in temperature and precipitation and monthly changes in dengue transmission in Puerto Rico. We also found that the strength of this association varies spatially, that this variation is associated with differences in local climate, and that this relationship is consistent with laboratory studies of the impacts of these factors on vector survival and viral replication. These results suggest the importance of temperature and precipitation in the transmission of dengue viruses and suggest a reason for their spatial heterogeneity. Thus, while dengue transmission may have a general system, its manifestation on a local scale may differ from global expectations.

## Author Summary

Dengue viruses are a major health problem throughout the tropical and subtropical regions of the world. Because they are transmitted by mosquitoes that are sensitive to changes in rainfall and temperature, transmission intensity may be regulated by weather and climate. Laboratory studies have shown this to be biologically plausible, but studies of transmission in real-life situations have been inconclusive. Here we demonstrate that increased temperature and rainfall are associated with increased dengue transmission in subsequent months across Puerto Rico. We also show that differences in local climate within Puerto Rico can explain local differences observed in the relationship between weather and dengue transmission. This finding is important because it suggests that the determinants of transmission occur on a local level such that although dengue viruses have a basically universal transmission cycle, changes in temperature or rainfall may have diverse local effects.

## Introduction

The dengue viruses are the most widely distributed and damaging arthropod-borne viruses (arboviruses) affecting humans. The viruses and their predominant mosquito vector, *Aedes aegypti*, are endemic to most of the tropical and subtropical regions of the world, where they cause seasonal epidemics of varying size. The seasonal nature of transmission may reflect the influence of climate on the transmission cycle. Increases in temperature and precipitation can lead to increased *Ae. aegypti* abundance by increasing their development rate, decreasing the length of reproductive cycles, stimulating egg-hatching, and providing sites for egg deposition [1],[2],[3],[4]. Higher temperature further abets transmission by shortening the incubation period of the virus in the mosquito [5].

Theoretical models of dengue transmission dynamics based on mosquito biology support the importance of temperature and precipitation in determining transmission patterns [6],[7], but empirical evidence has been lacking. On global scales, several studies have highlighted common climate characteristics of areas where transmission occurs [8],[9],[10]. Meanwhile, longitudinal studies of empirical data have consistently shown that temperature and precipitation correlate with dengue transmission but have not demonstrated consistency with respect to their roles [11],[12],[13],[14],[15],[16],[17],[18],[19],[20],[21],[22]. For example, cumulative monthly rainfall and mean temperature correlated positively with increased dengue transmission on the Andaman Sea side of Southern Thailand [17]. On the Gulf of Thailand side, however, it was the number of rainy days (regardless of quantity) and minimum temperature that associated positively with incidence. Another study, farther north, in Sukhothai, Thailand, found that temperature had a negative effect on dengue transmission [16]. This finding only makes biological sense at the upper temperature limits of *Ae. aegypti* survival, an uncommon condition during the study period. Other biologically suspect findings include a model for Selangor, Malaysia where lagged precipitation was a significant predictor of early wet season dengue, but did not associate with a significant change in *Ae. aegypti* abundance [12], the theoretical mechanism for precipitation increasing transmission. Though the vector and fundamental transmission cycle are similar in all endemic areas, the described relationships between transmission and weather are highly variable and, in some cases, make little biological sense. Some of the different findings may be attributed to underlying climate heterogeneity; local mosquito populations may be limited by different aspects of the environment depending on the conditions that they experience. However, local differences may also be attributed to over-fitting and incomplete statistical control for autocorrelation and collinearity.

Autocorrelation and collinearity pose significant challenges as they have important implications for regression models. Autocorrelation arises as a natural feature of infectious disease systems as the number of new infections relates closely to the number of recent infections. In longitudinal regression analysis this generally results in correlated residuals. Collinearity presents its own problem. Weather variables and dengue incidence are strongly seasonal, but seasonality alone does not imply a meaningful association, particularly when the effects may occur over lags of weeks or even months. The scale of seasonal variability in this system is so high that any lagged seasonal weather variable can account for a large proportion of the variation in dengue incidence.

Here we analyzed the association of temperature and precipitation with dengue transmission in each of 77 municipalities of Puerto Rico over a 20 year period using adaptive natural cubic splines to adjust for seasonal confounding [23]. The only excluded municipality was Culebra, a separate island where transmission is relatively sporadic due to a very small resident population. Figure S1 shows monthly temperature, precipitation, and dengue incidence for three example municipalities. We used a hierarchical statistical model to examine local associations over time and spatial heterogeneity in the estimated local associations [24]. At the first stage, within each municipality, we estimated the local short-term association between monthly variation in weather variables and monthly variation in dengue incidence while controlling for the smooth seasonal pattern of each covariate and reducing autocorrelation in the residuals. More specifically, we fitted municipality-specific Poisson regression models with monthly dengue incidence regressed on monthly average temperature or precipitation with a population offset and a natural cubic spline function of time. Because there are inherent delays between weather, its impact on mosquito populations, and their subsequent impact on transmission patterns, we used distributed lag models to assess effects of weather on dengue transmission up to 6 months later [25]. In the second stage, we estimated global association by averaging the short-term associations across municipalities and identified local climate characteristics that modify the local short-term associations. This stage of analysis allows us to characterize the spatial heterogeneity of the relationship between weather and dengue transmission.

## Methods

### Data

Clinically suspected dengue infections are reported to the surveillance system maintained by the Puerto Rico Department of Health and the Centers for Disease Control and Prevention. Though suspected and laboratory-confirmed cases generally correlate highly, we use suspected cases because they are more sensitive given that approximately 60% of reported cases have inadequate samples for a definitive diagnosis. Here we analyze monthly totals by municipality from July 1986 through December 2006. Municipal population data was obtained from the 1980 [26], 1990 [27], and 2000 [28] United States censuses. Estimates for median household income and the percentage of individuals living below the poverty line for each municipality are from the 2000 census [29].

Monthly mean maximum temperature, mean minimum temperature, and cumulative precipitation are simulated on 1 km^2^ grids from cross-validated spatial models of weather station data [30]. Monthly average temperature is the mean of the monthly maximum and minimum. Monthly values of each weather variable are aggregated at the municipal level as an average of all internal pixels weighted by pixel population size [31]. This weighting adjusts the average to better reflect climate in the areas where people live and dengue transmission occurs.

### Model

The number of reported dengue cases, *y*, at time *t* for each municipality is modeled as,
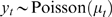


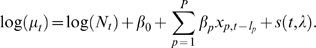



The population size, *N_t_*, is assumed to be a linear function of time parameterized by the 1980, 1990, and 2000 censuses [26],[27],[28]. The longitudinal covariates, *x_1_*,…, *x_P_*, are entered at covariate-specific distributed lags, *l_p_* [25]. The natural cubic spline smoothing function of time, *s(t, λ)*, is assigned *λ* of 2 degrees of freedom per year to fit a curve with a smooth seasonal pattern.

Parameter estimates, *β_p_* from each local regression model are compared using two-level normal independent sampling estimation [24]. First-stage location-specific (*j*) parameter estimates 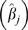 are distributed,
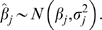



The variance, *σ_j_^2^*, can be estimated from first-level models. However, because the covariates are also modeled, and thus have estimated intrinsic variance, we repeated the regression model for each of 1,000 weather model conditional simulations and use the distribution of these 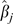 to estimate *σ_j_^2^*. Finally we added effect modifiers *z_1_*,…, *z_Q_*, to estimate the average effects (*α_0_*) and effect modification (*α_q_*) in a Bayesian model,
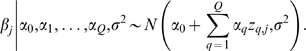



All analyses were performed in R (R Foundation for Statistical Computing, Vienna, Austria, 2007).

## Results

**Figure 1:**
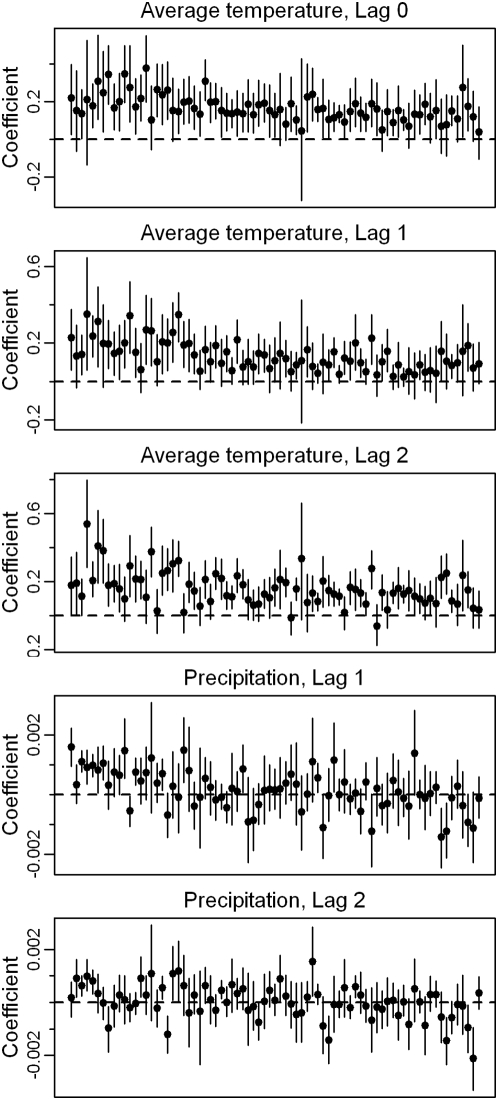
Local short-term associations between weather and dengue incidence. Points represent the estimated proportional increase in dengue incidence for an increase in monthly temperature (1°C) or precipitation (1 mm) in each municipality at each lag (months). All lagged weather variables were included in the regression model simultaneously. Municipalities are ordered by mean average temperature or precipitation, low to high (left to right). Black bars indicate the 95% credible interval for each estimate based on 1,000 models, one for each conditional simulation of weather data.

In the distributed lag model including temperature at lags of 0, 1, and 2 months and precipitation at lags of 1 and 2 months, monthly variation in temperature was positively associated with monthly variation in dengue incidence in most municipalities (Figure 1). The global association (average short-term association across all municipalities) was positive and statistically significant at all three lags. Short-term associations were significant for monthly maximum and minimum temperature but weaker than those observed for average monthly temperature. Monthly variation in cumulative precipitation was significantly associated with monthly variation in dengue incidence in some, but not all, municipalities at lags of 1 and 2 months (Figure 1). Globally, this association was significantly positive only after accounting for local effect modification by long-term climate. These findings were robust to the addition of further temperature and precipitation lags.

Long-term climate variables modified the local short-term association between monthly weather and dengue incidence for all variables at all lags. Long-term mean temperature significantly modified the short-term effect of monthly temperature and long-term mean precipitation significantly modified the effect of short-term precipitation. In municipalities with higher long-term temperature or precipitation, the short-term association between temperature or precipitation, respectively, and dengue incidence was weaker. For instance, the model predicts that an area with long-term mean temperature of approximately 30°C will exhibit no significant association between monthly variations in temperature and dengue incidence. This threshold was consistent across lags (30.6°C, 30.2°C, and 29.9°C for 0, 1, and 2 month lags, respectively) and is compatible with laboratory studies which indicate that dengue transmission is optimized at temperatures above 30°C [5]. The effect of monthly precipitation is likewise minimized where mean annual precipitation is high, approximately 1,800 mm in Puerto Rico (1,820 mm and 1,760 mm, for 1 and 2 month lags, respectively). This condition is present in some areas of Puerto Rico and explains the lack of significant association between precipitation and dengue incidence in some municipalities. These thresholds must be considered as specific to Puerto Rico because they are contingent upon regional climate. High annual precipitation, for instance, may indicate consistently high precipitation or alternatively, a brief period of intense precipitation. In the latter case, even an area with high annual precipitation may exhibit a strong association with dengue transmission on the monthly scale.

In addition to long-term mean climate measures, we analyzed effect modification associated with socio-economic factors including population density, median household income, and the percentage of families living below the poverty line. In municipalities with a higher poverty index the short-term association between weather variables and dengue incidence was stronger, but this effect was not consistent across lags.

**Figure 2:**
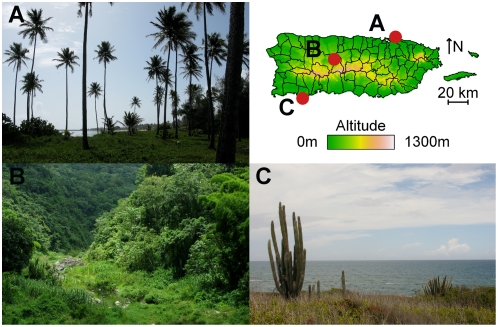
Elevation and climate variation in Puerto Rico. The map shows the location of each photograph. Natural areas of northern Puerto Rico are moist subtropical forest. Higher elevations are subtropical or montane wet or rain forests. The southwestern coast is subtropical dry forest. Figure S1 shows monthly temperature and precipitation in proximal areas.

**Figure 3:**
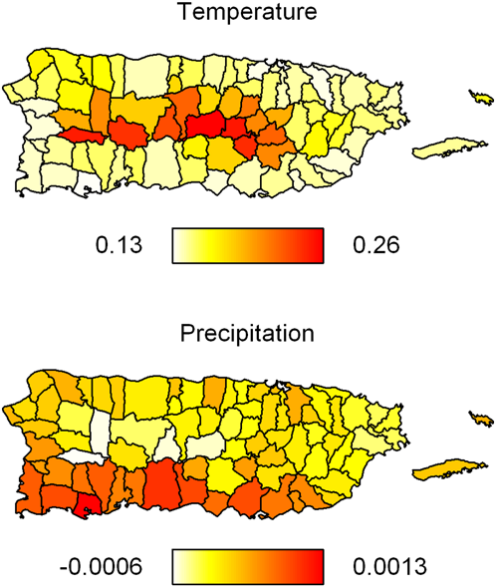
Spatial effects of climate on dengue incidence. Colors represent the relative strength of the cumulative association between monthly temperature and precipitation in each municipality. This is calculated as the cumulative effect of a 1°C increase in mean monthly temperature on dengue incidence in the current and two subsequent months and of a 1 mm increase in precipitation on dengue incidence in the two following months.

For a small island, Puerto Rico contains remarkable climate diversity: the northeastern coastal area is warm and wet; the central mountains, cooler and wetter; and the southwestern coastal region, hot and dry (Figure 2, Figure S1). The effects observed here demonstrate how these differences influence the association between weather and dengue transmission. Figure 3 shows the cumulative effects of an increase in monthly temperature and precipitation on dengue incidence in the same month and in the following 1 to 2 months in each municipality. The cumulative effects were obtained by summing the short-term associations estimated at each lag. As expected, the cumulative effect of temperature on dengue incidence is highest in the cooler mountainous areas. Likewise, the role that precipitation plays is greatest in the dry southwestern coastal region. Regional patterns in transmission may result from both from shared characteristics and from the movement of infected humans or vectors among proximal municipalities. The spatial patterns observed in Figure 3 demonstrate regional behaviour due to climate. Though there may be additional effects of proximity, those described here are robust as they are derived based solely on local characteristics and only later compared at the global scale. Analysis of further spatial correlation due to movement requires more detailed mechanistic models beyond the scope of the current analysis.

## Discussion

The associations between temperature, precipitation, and dengue transmission reported here are strong and consistent through time. Moreover, these associations depend on local characteristics and have a biological interpretation. Together these associations suggest an important relationship. It is critical, however, to consider the extent of the role which temperature and precipitation may play in increasing dengue incidence. The spline smooth in the current analysis reduces the extensive inter-annual variation in incidence observed in endemic areas like Puerto Rico such that the analysis effectively isolates association on finer, monthly, temporal scales. Thus, while we have reported a significant association between climate and dengue incidence, it is on a month-to-month time scale and does not show that warmer years (for example) consistently exhibit higher overall incidence. Though temperature and precipitation may also influence the magnitude of yearly transmission, this analysis does not demonstrate that. Studies on the relationship between multi-year climate variation and dengue incidence are inconsistent and at most account for only part of inter-annual variation in dengue transmission [11],[21],[32],[33],[34],[35],[36] (Puerto Rico manuscript in preparation; M.A.J., D.A.T. Cummings, & G.E.G.). Cogent alternative hypotheses suggest the importance of intrinsic factors related to the interactions of the four serotypes of dengue virus with human populations [37],[38],[39],[40].

The spline itself, being seasonal by definition, likely contains more variation that in reality is attributable to weather. Removing this variation from the analysis is critical to differentiating the effects of the covariates which also exhibit smooth seasonal trends. This makes the associations evident but may underestimate the magnitude of the true effects of temperature and precipitation. As described above in terms of inter-annual variation, the spline makes forecasting even on the monthly scale problematic. Although this limits the public health utility of our findings, the empirical demonstration of these associations and their dependence on the underlying climate are important for the understanding of transmission dynamics and the potential effects of changing climate. Our findings suggest that in areas where temperature and precipitation are already high, increases in either will have little effect on transmission.

Spatial heterogeneity in transmission is a common feature of vector-borne pathogens and many other infectious diseases. Because of this, transmission may be more completely described by research focused on a local scale. However, such studies lack generalizability as the factors limiting transmission may not be universal. Large scale hierarchical studies containing spatial heterogeneity and reasonable consideration of confounding have the potential to reveal universal underlying patterns while simultaneously describing unique local conditions. The two-stage analysis employed here to estimate average global effects and determinants of local variation is an approach that could be applied to a number of other environmentally-mediated diseases. Previous studies of the empirical relationship between weather and local dengue transmission highlighted local differences and identified global climate characteristics of areas where transmission has been reported. Here, for the first time, we link the local, temporal relationship between weather and dengue transmission to underlying climate characteristics to account for heterogeneity in local transmission patterns.

## Supporting Information

Figure S1 Local time series. Monthly dengue incidence, temperature, and precipitation for 3 municipalities. San Juan is a large urban municipality on the North coast near photo A in Figure 2. Adjuntas is a small rural municipality in the central mountains near photo B in Figure 2. Ponce is a large mostly-urban municipality on the South coast near photo C in Figure 2. Temperature and precipitation represent the range of values produced in the weather models. High variability in predicted precipitation is evident.
(0.04 MB PDF)

## References

[1] Christophers SR (1960). *Aedes aegypti* (L.): The Yellow Fever Mosquito.

[2] Keirans JE, Fay RW (1968). Effect of food and temperature on *Aedes aegypti* (L.) and *Aedes triseriatus* (Say) larval development.. Mosq News.

[3] Pant CP, Yasuno M (1973). Field studies on the gonotrophic cycle of *Aedes aegypti* in Bangkok, Thailand.. J Med Entomol.

[4] Rueda LM, Patel KJ, Axtell RC, Stinner RE (1990). Temperature-dependent development and survival rates of *Culex quinquefasciatus* and *Aedes aegypti* (Diptera: Culicidae).. J Med Entomol.

[5] Watts DM, Burke DS, Harrison BA, Whitmire RE, Nisalak A (1987). Effect of temperature on the vector efficiency of Aedes aegypti for dengue 2 virus.. Am J Trop Med Hyg.

[6] Bartley LM, Donnelly CA, Garnett GP (2002). The seasonal pattern of dengue in endemic areas: mathematical models of mechanisms.. Trans R Soc Trop Med Hyg.

[7] Hopp MJ, Foley JA (2003). Worldwide fluctuations in dengue fever cases related to climate variability.. Climate Research.

[8] Hales S, de Wet N, Maindonald J, Woodward A (2002). Potential effect of population and climate changes on global distribution of dengue fever: an empirical model.. Lancet.

[9] Peterson AT, Martinez-Campos C, Nakazawa Y, Martinez-Meyer E (2005). Time-specific ecological niche modeling predicts spatial dynamics of vector insects and human dengue cases.. Transactions of the Royal Society of Tropical Medicine and Hygiene.

[10] Rogers DJ, Wilson AJ, Hay SI, Graham AJ (2006). The global distribution of yellow Fever and dengue.. Advances in Parasitology.

[11] Hay SI, Myers MF, Burke DS, Vaughn DW, Endy T (2000). Etiology of interepidemic periods of mosquito-borne disease.. Proc Natl Acad Sci U S A.

[12] Foo LC, Lim TW, Lee HL, Fang R (1985). Rainfall, abundance of Aedes and dengue infection in Selangor, Malaysia.. Southeast Asian J Trop Med Pub Health.

[13] Keating J (2001). An investigation into the cyclical incidence of dengue fever.. Soc Sci Med.

[14] Schreiber KV (2001). An investigation of relationships between climate and dengue using a water budgeting technique.. Int J Biometeorol.

[15] Depradine C, Lovell E (2004). Climatological variables and the incidence of Dengue fever in Barbados.. Int J Environ Health Res.

[16] Nakhapakorn K, Tripathi NK (2005). An information value based analysis of physical and climatic factors affecting dengue fever and dengue haemorrhagic fever incidence.. Int J Health Geogr.

[17] Promprou S, Jaroensutasinee M, Jaroensutasinee K (2005). Climatic Factors Affecting Dengue Haemorrhagic Fever Incidence in Southern Thailand.. Dengue Bulletin.

[18] Chowell G, Sanchez F (2006). Climate-based descriptive models of dengue fever: the 2002 epidemic in Colima, Mexico.. J Environ Health.

[19] de Souza IC, Vianna RP, de Moraes RM (2007). [Modeling of dengue incidence in Paraíba State, Brazil, using distributed lag models].. Cad Saude Publica.

[20] Wu PC, Guo HR, Lung SC, Lin CY, Su HJ (2007). Weather as an effective predictor for occurrence of dengue fever in Taiwan.. Acta Trop.

[21] Hurtado-Díaz M, Riojas-Rodríguez H, Rothenberg SJ, Gomez-Dantés H, Cifuentes E (2007). Short communication: impact of climate variability on the incidence of dengue in Mexico.. Trop Med Int Health.

[22] Rosa-Freitas MG, Schreiber KV, Tsouris P, Weimann ET, Luitgards-Moura JF (2006). Associations between dengue and combinations of weather factors in a city in the Brazilian Amazon.. Rev Panam Salud Publica.

[23] Hastie TJ, Chambers JM, Hastie TJ (1992). Generalized additive models.. Statistical Models in S. Boca Raton.

[24] Everson PJ, Morris CN (2000). Inference for Multivariate Normal Hierarchical Models.. Journal of the Royal Statistical Society, Series B.

[25] Welty LG, Peng RD, Zeger SL, Dominici F (2008). Bayesian Distributed Lag Models: Estimating Effects of Particulate Matter Air Pollution on Daily Mortality.. Biometrics Epub.

[26] (1984). 1980 Census of Population. Vol. 1: Characteristics of the Population. Part 53A: Puerto Rico..

[27] (1991). 1990 Census of Population: General Population Characteristics: Puerto Rico..

[28] (2001). Census 2000 Summary File 1 Puerto Rico..

[29] (2002). Census 2000 Summary File 3 Puerto Rico..

[30] Johansson MA, Glass GE (2008). High-resolution spatiotemporal weather models for climate studies.. International Journal of Health Geographics.

[31] (2004). LandScan Global Population Database.. Oak Ridge National Laboratory.

[32] Hales S, Weinstein P, Woodward A (1996). Dengue fever epidemics in the South Pacific: driven by El Niño Southern Oscillation?. Lancet.

[33] Hales S, Weinstein P, Souares Y, Woodward A (1999). El Niño and the dynamics of vectorborne disease transmission.. Environ Health Perspect.

[34] Gagnon AS, Bush ABG, Smoyer-Tomic KE (2001). Dengue epidemics and the El Niño Southern Oscillation.. Climate Research.

[35] Cazelles B, Chavez M, McMichael AJ, Hales S (2005). Nonstationary influence of El Niño on the synchronous dengue epidemics in Thailand.. PLoS Med.

[36] Bangs MJ, Larasati RP, Corwin AL, Wuryadi S (2006). Climatic factors associated with epidemic dengue in Palembang, Indonesia: implications of short-term meteorological events on virus transmission.. Southeast Asian J Trop Med Public Health.

[37] Ferguson N, Anderson R, Gupta S (1999). The effect of antibody-dependent enhancement on the transmission dynamics and persistence of multiple-strain pathogens.. Proc Natl Acad Sci U S A.

[38] Schwartz IB, Shaw LB, Cummings DA, Billings L, McCrary M (2005). Chaotic desynchronization of multistrain diseases.. Phys Rev E.

[39] Wearing HJ, Rohani P (2006). Ecological and immunological determinants of dengue epidemics.. Proc Natl Acad Sci U S A.

[40] Adams B, Holmes EC, Zhang C, Mammen MP, Nimmannitya S (2006). Cross-protective immunity can account for the alternating epidemic pattern of dengue virus serotypes circulating in Bangkok.. Proc Natl Acad Sci U S A.

