# Supplementary material for: Local and Global Effects of Climate on Dengue Transmission in Puerto Rico
Source: PLoS Negl Trop Dis. 2009 Feb 17;3(2):e382. doi: 10.1371/journal.pntd.0000382 (PMC2637540; doi:10.1371/journal.pntd.0000382)
Supplement: Figure S1 — Local time series. Monthly dengue incidence, temperature, and precipitation for 3 municipalities. San Juan is a large urban municipality on the North coast near photo A in Figure 2. Adjuntas is a small rural municipality in the central mountains near photo B in Figure 2. Ponce is a large mostly-urban municipality on the South coast near photo C in Figure 2. Temperature and precipitation represent the range of values produced in the weather models. High variability in predicted precipitation is evident. (0.04 MB PDF) [file pntd.0000382.s001.pdf]

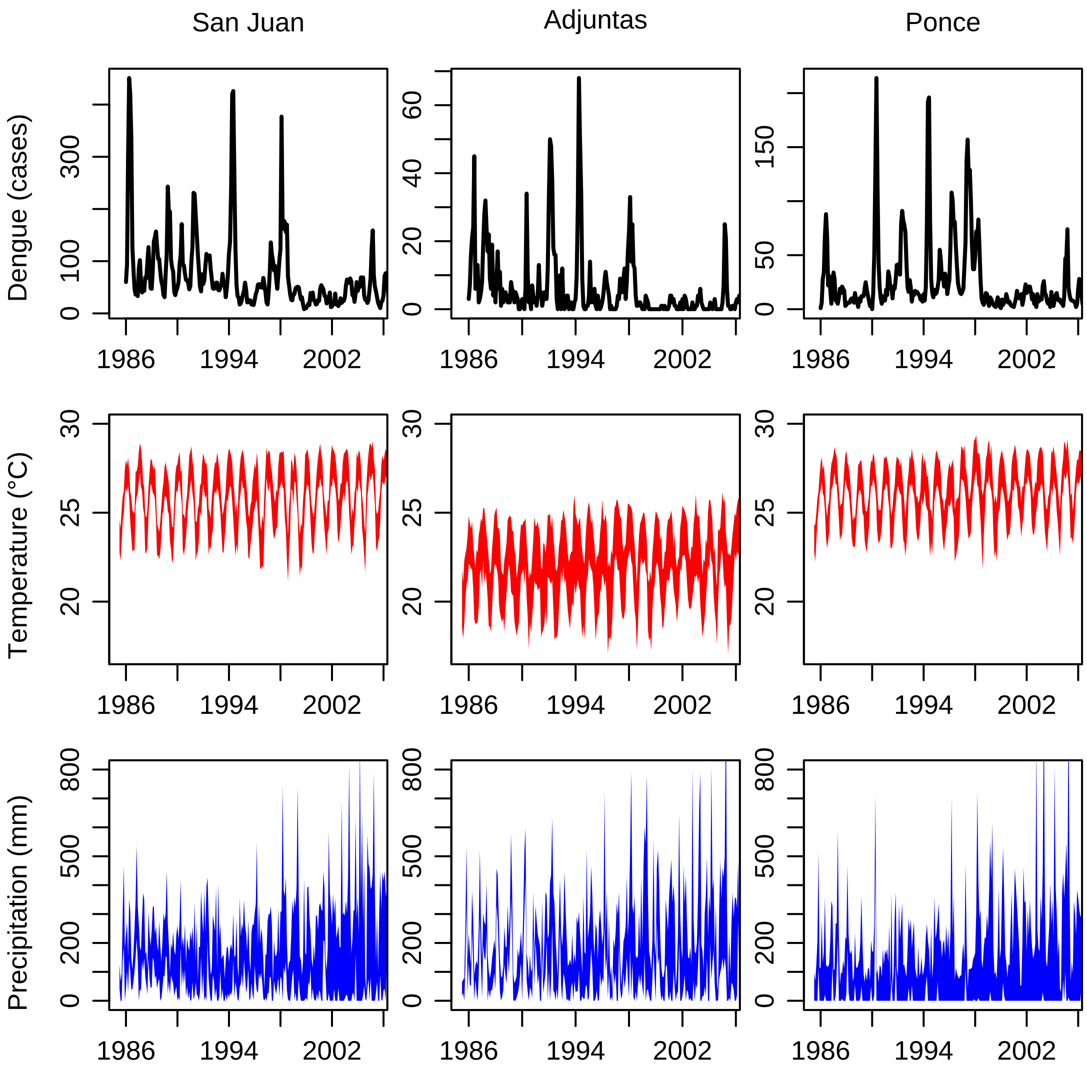

Figure S1. Local time series. Monthly dengue incidence, temperature, and precipitation for 3 municipalities. San Juan is a large urban municipality on the North coast near photo **A** in Figure 2. Adjuntas is a small rural municipality in the central mountains near photo **B** in Figure 2. Ponce is a large mostly-urban municipality on the South coast near photo **C** in Figure 2. Temperature and precipitation represent the range of values produced in the weather models. High variability in predicted precipitation is evident.
